# Microbial nitrogen fixation and methane oxidation are strongly enhanced by light in *Sphagnum* mosses

**DOI:** 10.1186/s13568-020-00994-9

**Published:** 2020-03-31

**Authors:** Martine A. R. Kox, Eva van den Elzen, Leon P. M. Lamers, Mike S. M. Jetten, Maartje A. H. J. van Kessel

**Affiliations:** 1 Department of Microbiology, Radboud University, Heijendaalseweg 135, 6525 AJ Nijmegen, The Netherlands; 2 Department of Aquatic Ecology and Environmental Biology, Radboud University, Heijendaalseweg 135, 6525 AJ Nijmegen, The Netherlands

**Keywords:** Methanotrophy, Diazotrophy, *Sphagnum* moss, Peatland, Light

## Abstract

Peatlands have acted as C-sinks for millennia, storing large amounts of carbon, of which a significant amount is yearly released as methane (CH_4_). *Sphagnum* mosses are a key genus in many peat ecosystems and these mosses live in close association with methane-oxidizing and nitrogen-fixing microorganisms. To disentangle mechanisms which may control *Sphagnum*-associated methane-oxidation and nitrogen-fixation, we applied four treatments to *Sphagnum* mosses from a pristine peatland in Finland: nitrogen fertilization, phosphorus fertilization, CH_4_ addition and light. N and P fertilization resulted in nutrient accumulation in the moss tissue, but did not increase *Sphagnum* growth. While net CO_2_ fixation rates remained unaffected in the N and P treatment, net CH_4_ emissions decreased because of enhanced CH_4_ oxidation. CH_4_ addition did not affect *Sphagnum* performance in the present set-up. Light, however, clearly stimulated the activity of associated nitrogen-fixing and methane-oxidizing microorganisms, increasing N_2_ fixation rates threefold and CH_4_ oxidation rates fivefold. This underlines the strong connection between *Sphagnum* and associated N_2_ fixation and CH_4_ oxidation. It furthermore indicates that phototrophy is a strong control of microbial activity, which can be directly or indirectly.

## Introduction

The large amounts of carbon (C) stored in Northern peatlands (representing 250–450 Pg C; Frolking and Roulet 2007) are severely threatened by anthropogenic disturbances of these vulnerable ecosystems due to for example drainage, fires and N-fertilization (Turetsky et al. 2002; Bragazza et al. 2012; Andersen et al. 2013; Abdalla et al. 2016; Leifeld and Menichetti 2018). Over millennia peatlands have acted as net C-sinks, sequestering and storing more C than is emitted to the atmosphere, thereby counteracting global warming (Gorham 1991; Rydin and Jeglum 2006; Frolking and Roulet 2007; Loisel et al. 2014; Leifeld and Menichetti 2018). C is mainly stored as dead organic matter (Loisel et al. 2014) originating moss and plants such as from *Sphagnum* mosses, *Carex* species that dominate many of these peatlands.

Most *Sphagnum* species thrive in nitrogen (N) limited environments (Aerts et al. 1992) and do so by monopolizing the majority of atmospheric N deposited (Fritz et al. 2014). *Sphagnum* has a high affinity for N (K_s_ 3.5–6.5 μM) and will rapidly take up ammonium or nitrate when available (Fritz et al. 2014). In addition to the limited pool of environmentally available N, N required for growth is also provided by microbial N_2_ fixation via *Sphagnum*-associated microorganisms, which may account for the mismatch between the high N-content of *Sphagnum* mosses and the low input of N via atmospheric deposition (Vile et al. 2014). In younger peatlands, N_2_ fixation by methane-oxidizing microorganisms is assumed to be responsible for N accumulation in *Sphagnum* (Larmola et al. 2014), providing a strong link between the CH_4_-cycle and N-cycle in *Sphagnum*-dominated peatlands (Ho and Bodelier 2015).

Worldwide, the N-cycle in peatlands has been disturbed by long-term, anthropogenic N fertilization. This has reduced C-accumulation rates and altered decomposition of stored organic matter (Bragazza et al. 2012) and ultimately leads to changes in the plant-community composition (Tomassen et al. 2004; Fritz et al. 2012). As N-fertilization skews the N:phosphorus (P) ratio in *Sphagnum* mosses, it was thought that additional P-fertilization might alleviate the effect of N-fertilization. However, this appeared to be only partially true, as mosses remained N-saturated and experienced N-stress despite P-fertilization (Fritz et al. 2012). It is, however, clear that the N-fertilization effect is strongly linked to nutrient stoichiometry, especially in relation to P-availability. Excess N and disturbance of the N:P ratio can greatly affect the delicate balance between C-sink and C-source in peatlands (Turetsky et al. 2002; Frolking et al. 2011; Kivimäki et al. 2013).

The response of the nitrogen-fixing microbial community associated with *Sphagnum* mosses to N-fertilization remains puzzling. Short-term high N-fertilization has been shown to result in reduced N_2_ fixation activity in peatland *Sphagnum* mosses (Kox et al. 2016) as well as in forest mosses (Leppänen et al. 2013). Yet, long-term N-fertilization has been shown to hardly affect N_2_ fixation in *Sphagnum* (Kox et al. 2016; van den Elzen et al. 2018). P-fertilization, on the other hand appears to stimulate N_2_ fixation activity of the associated microorganisms, but not necessarily *Sphagnum* moss growth (Fritz et al. 2012; van den Elzen et al. 2017; Rousk et al. 2017).

The N-fertilization effect on CH_4_ oxidation activity depends largely on land use, and the extent and duration of fertilization (Veraart et al. 2015). For bog ecosystems, long term fertilization with N, P and potassium (K) fertilization resulted in increased CH_4_ emission, mostly via indirect effects such as changes in vegetation structure and peat properties (Juutinen et al. 2018). However, P and K rather than N fertilization seemed to have contributed to this result, since the PK-only treatment of the study stimulated methanogenesis, whereas N fertilization did not affect CH_4_ production or consumption (Juutinen et al. 2018).

Next to N-fertilization, light intensity and its diurnal rhythm do affect C-sequestration by *Sphagnum* mosses (Laine et al. 2011; Kangas et al. 2014). Different *Sphagnum* species are adapted to different light and moisture conditions, which is reflected in their productivity (Kangas et al. 2014; Bengtsson et al. 2016). The diurnal rhythm of *Sphagnum* mosses influences the conditions in and around the mosses. Although there is little specific literature available on this diurnal effect on the *Sphagnum* microbiome, it is highly likely that the daily light regime affects the *Sphagnum*-associated microbiome (Larmola et al. 2014; van den Elzen et al. 2017).

In this study, the primary goal was to investigate the environmental drivers of N_2_ fixation and CH_4_ oxidation in pristine *Sphagnum* mosses. To this end, peat sods were collected from a pristine site in Northern Finland. Fertilization (control, N, P or N + P), CH_4_, and light treatments were applied in a full factorial set-up to further disentangle the mechanisms controlling *Sphagnum*-associated CH_4_ oxidation and N_2_ fixation. It was expected that N fertilization would result in decreased N_2_ fixation activity, whereas P fertilization would show increased N_2_ fixation rates but not necessarily CH_4_ oxidation rates. Both N_2_ fixation and CH_4_ oxidation rates were expected to be higher in light conditions. CH_4_ addition on the other hand was expected to stimulate methane-oxidizing nitrogen-fixing microorganisms. Moss growth was expected to benefit from any type of fertilization as the mosses were collected from a pristine peat site and thus nutrient limited.

In a climate-controlled room, the peat sods received N, P, both N + P fertilization or no fertilization at all (control) and received additional or no additional CH_4_. In addition, all measurements were performed in light and dark to test the influence of light. Both in the field and in the lab, we measured net gas fluxes of CO_2_ and CH_4_ in dark and light, and determined rates of microbial N_2_ fixation and CH_4_ oxidation rates in the dark and light using stable isotopes.

## Materials and methods

### Sampling site

The sampling site was located in Siikajoki, Finland, a well-studied pristine peatland [N deposition rate locally 0.3 g m^−2^ year^−1^; Mustajärvi et al. (2008)] located in the middle of the boreal ecoclimatic zone (64° 45′ N, 24° 42′ E; Tuittila et al. 2013; Larmola et al. 2014). At an oligotrophic fen site SJ4 (Laine et al. 2011) eight peat sods dominated by *S. papillosum* were collected (50 × 25 × 20 cm; l × w × h) and four 20 L vessels with peat water were collected and transported back to Nijmegen, the Netherlands, within 48 h. Upon arrival in the laboratory, each peat sod was cut into 4 mesocosms which were immediately placed into glass aquaria (25 × 12 × 30 cm; l × w × h), creating 32 mesocosms (25 × 12 × 20 cm; l × w × h). The water level was maintained at 10 cm below the moss surface, which was similar to the natural situation, using the peat water collected locally.

Mesocosms were kept at 15 °C by means of a water bath in a climate chamber (average 24 °C), and exposed to a 16 h light period per day (Philips greenpower LED, Poland) providing approximately 800 µmol PAR m^−2^ s^−1^. Mesocosms were acclimatized in the climate chamber for 6 weeks prior to experimental treatment.

### Experimental design

Incubations for the determination of N_2_ fixation and CH_4_ oxidation rates, both in dark and light conditions, were performed. In the lab, a full factorial set-up was used to study the effect of fertilization, CH_4_ addition and light as specified in Table 1. Each treatment had 4 replicates, resulting in 32 individual mesocosms; samples originating from one sod did not get the same treatment. The fertilization treatments consisted of artificial rainwater Kox et al. (2016) with additional N fertilization (25 kg N ha^−1^ year^−1^ as NH_4_NO_3_), P fertilization (10 kg P ha^−1^ year^−1^ as KH_2_PO_4_), or a combination of both (NP). The non-fertilized controls (C) received limited background levels of N and P (resp. 0.5 kg N ha^−1^ year^−1^ as NH_4_NO_3_ and 0.2 kg P ha^−1^ year^−1^ as KH_2_PO_4_). N fertilization was applied by sprinkling the fertilized rainwater on top of the moss layer, whereas P fertilization was supplied via parallel injections (25 times) 1.5 cm below the moss surface mimicking subsurface P fertilization. For the methane treatment, mesocosms received methane (1.3 mmol CH_4_ L^−1^) dissolved in artificial rainwater, via the bottom of the mesocosm once a week (1 L). In the control treatment, dissolved Argon was added instead of CH_4_. The amount of rainwater provided was equalized to the mean annual rainfall in Northern Finland [521 mm year^−1^; Drebs et al. (2002)]. The experiment lasted for 10 weeks.

**Table 1:** Overview of the experimental set-up with CH_4_ addition and fertilization treatments

Treatment	CH_4_	Concentration N (kg ha^−1^ year^−1^)	Concentration P (kg ha^−1^ year^−1^)	n
Control	No	0.5	0.2	4
N	No	25	0.2	4
P	No	0.5	10	4
N + P	No	25	10	4
Control + CH_4_	Yes	0.5	0.2	4
N + CH_4_	Yes	25	0.2	4
P + CH_4_	Yes	0.5	10	4
N + P + CH_4_	Yes	25	10	4

Effects of light/dark were measured in light or dark periods in the climate room

### Porewater and moss chemistry

Porewater samples were collected via Rhizons (0.2 µm; 5 cm length in the field; Eijkelkamp Agrisearch Equipment, Giesbeek, the Netherlands). Porewater pH, alkalinity, concentrations of PO_4_^3−^, NO_3_^−^, NH_4_^+^ and elemental composition were analyzed as described by Van den Elzen et al. (2017). DOC and TN were determined by combustion (Shimadzu, Duisburg, Germany). Upper 3 cm of three *Sphagnum* specimens were sampled for total N, P and K concentrations in moss tissue, which was analyzed as described by Van den Elzen et al. (2017). All data is presented in Additional file 1: Table S1.

### Total gas flux analysis

In the mesocosms, total fluxes of CO_2_ and CH_4_ in light and dark were measured using a Picarro G2508 Greenhouse Gas Analyzer with cavity ringdown spectroscopy (Picarro Inc., Santa Clara, CA, USA). When measuring fluxes, mesocosms were closed air-tight using a lid and paste (Terostat IX, Teroson GmbH, Heidelberg, Germany). Temperature and light conditions were logged using a HOBO pendant temperature and light logger (Onset, Bourne, MA, USA). Fluxes are expressed as mg m^−2^ h^−1^ for measured rates, but as mg m^−2^ day^−1^ for diurnal fluxes, calculated based on 8 h dark and 16 h light regime.

### Moss performance

In each mesocosm 2 sticks were placed and marked to determine the height increase of the moss surface. *Sphagnum* surface height increase relative to each stick was recorded every 2 weeks.

## ^15^N_2_ fixation and ^13^CH_4_ oxidation rates

For each activity assay, mosses (top 3 cm length) were carefully collected from each mesocosm. Per incubation three moss parts were put in a 120 ml serum vial, closed with a butyl rubber stopper and crimp capped. To determine nitrogen fixation activity, batch incubations were supplied with 15% labelled ^15^N–N_2_. Another set of incubations received ^13^C-labeled CH_4_ (5%) which was additionally to the 15% labelled ^15^N–N_2_ to determine CH_4_ oxidation potential. Incubations were kept for 48 h at 24 °C either in the light or in the dark. After incubation, mosses were oven-dried for 48 h at 70 °C and dried material was ground using a mixer mil (MM301, Retsch, Germany) for 2 min at 30 rotations s^−1^. To determine the fraction of ^15^N or ^13^C, plant biomass was taken (resp. 5 and 0.225 mg) in duplicates and analyzed for ^15^N and ^13^C content using an elemental (CNS) analyzer coupled to an Isotopic Ratio Mass Spectrometer as described in Kox et al. (2018).

### Statistical analysis

Data was analyzed using R version 3.4.0 (R Development Core Team 2017). Each treatment consisted of 4 replicates (Table 1). Normality of data was tested using Shapiro–Wilk’s test on the residual (stats-package) and homogeneity of variance was tested using Levene’s test (car-package). Data following a normal distribution were analyzed using ANOVA followed by Tukey HSD post hoc test. In case of non-normal or heteroscedastic data, data was transformed prior to analysis. This was the case for N-content (1/n transformation), CO_2_ flux and CH_4_ flux in both light and dark conditions (log transformation), ^15^N–N_2_ fixation (log transformed), ^13^C–CH_4_ oxidation (log-transformed). Data whose distribution remained unaffected upon transformation (C-content, P-content, Porewater) were analyzed using non-parametric tests (Kruskal–Wallis followed by Dunn test).

## Results

Here we investigate the environmental drivers of microbial N_2_ fixation and CH_4_ oxidation in pristine *Sphagnum* mosses. Fertilization treatment (Control, N, P, and NP) as well as addition of CH_4_ and effect of light were applied to further disentangle the mechanisms controlling *Sphagnum*-associated CH_4_ oxidation and N_2_ fixation.

### CH_4_ addition effect

The weekly addition of dissolved CH_4_ did not significantly change the overall concentration of dissolved CH_4_ in the mesocosms (CH_4_ addition 510 ± 42 μmol L^−1^ (mean ± SEM); no-addition 471 ± 31 μmol L^−1^; Additional file 1: Figure S1, F_1,24_ = 0.5, p > 0.05), indicating that sufficient endogenous methane was being produced during the 10 weeks of incubation. Therefore, the results from the CH_4_ addition and control without methane additions mesocosms were merged in the further analysis, which results in an increase of the number of replicates (resulting in a duplication of samples from 4 to 8, as also indicated in Tables 4, 5 and 6) for each fertilization treatment and light or dark treatment.

### NP fertilization effect

The mosses grew on average 0.43 ± 0.02 mm day^−1^ (equaling 3 cm in 10 weeks; Table 2). Moss growth was not affected by 10 weeks of N, P or N + P fertilization (p > 0.05). C content of the mosses (range 434–444 mg C g^−1^ dry weight (DW), Table 3) was not affected by the fertilization treatments (χ^2^(3) = 1.6, p > 0.05), and neither were porewater dissolved organic carbon (DOC, 86–110 ppm) and total N (TN, 1.7–1.9 ppm) (Table 4; DOC F_3,28_ = 0.82, p > 0.05; TN F_3,28_ = 0.36, p > 0.05).

**Table 2:** *Sphagnum* moss growth (mm day^−1^) for each treatment

Treatment	Moss growth (mm day^−1^)		n^a^
	Mean ± SEM		
Control	0.47 ± 0.05	*ns*	8
N	0.38 ± 0.03	*ns*	8
N + P	0.46 ± 0.03	*ns*	8
P	0.42 ± 0.02	*ns*	8

*ns* not significant

^a^CH_4_ addition treatment was merged with its control, resulting in doubling of the replicates

**Table 3:** C, N, P, and K contents of *Sphagnum* moss tissue

Treatment	C content (mg C g^−1^)		N content (mg N g^−1^)		P content (mg P g^−1^)		K content (mg K g^−1^)		n^a^
	Mean ± SEM		Mean ± SEM		Mean ± SEM		Mean ± SEM		
Control	444 ± 2.9	*ns*	7.2 ± 0.1	*a*	0.49 ± 0.02	*a*	7.6 ± 0.3	*ac*	32
N	440 ± 2.0	*ns*	8.1 ± 0.3	*b*	0.47 ± 0.01	*a*	6.4 ± 0.2	*b*	32
N + P	434 ± 10	*ns*	7.8 ± 0.2	*ab*	0.57 ± 0.02	*b*	6.5 ± 0.2	*ab*	32
P	443 ± 2.0	*ns*	7.4 ± 0.2	*a*	0.50 ± 0.04	*a*	8.3 ± 0.5	*c*	32

For each measured element the differences between treatments are indicated with italic letters, identical letters indicate no difference

*ns* not significant

^a^As n was the same for each measurement it is only mentioned once here. CH_4_ addition treatment was merged with its control, resulting in doubling of the replicates

**Table 4:** Porewater DOC and TN content (ppm) for each treatment

Treatment	DOC (ppm)			TN (ppm)		
	Mean ± SEM	n^a^		Mean ± SEM	n^a^	
Control	110 ± 14	8	*ns*	1.91 ± 0.15	8	*ns*
N	93 ± 13	8	*ns*	1.71 ± 0.15	8	*ns*
N + P	104 ± 10	8	*ns*	1.87 ± 0.16	8	*ns*
P	86 ± 9.3	8	*ns*	1.76 ± 0.17	8	*ns*

*ns* not significant

^a^CH_4_ addition treatment was merged with its control, resulting in doubling of the replicates

The N content of N-fertilized moss (Table 3; N-fertilized 8.1 ± 0.3 mg N g^−1^; NP fertilized 7.8 ± 0.2 mg N g^−1^) was about 10% higher (F_3,123_ = 4.6, p < 0.005) than those of the control and P fertilized plants (control 7.2 ± 0.1 mg N g^−1^; P fertilized 7.4 ± 0.2 mg N g^−1^). K-content of the moss biomass showed the opposite pattern, with N fertilized mosses containing 18% less K (F_3,123_ = 7.87, p < 0.001) compared to non-N-fertilized treatments (Table 3). Analysis of the moss P content revealed that NP fertilized mosses contained 16% more P than all other treatments (Table 3, χ^2^(3) = 21.7, p < 0.001).

#### CO_2_ and CH_4_ fluxes

**Fig. 1:**
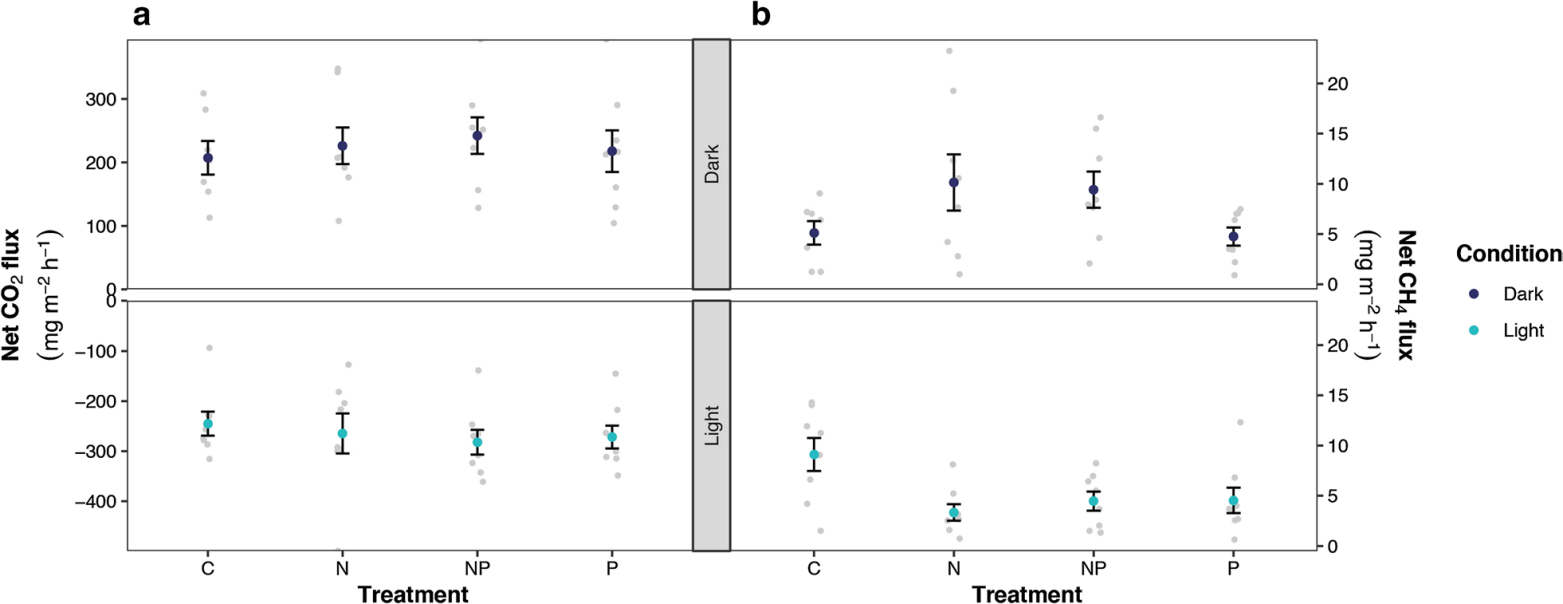
Plot showing the average **a** net CO_2_ flux (mg C m^−2^ h^−1^) and **b** net CH_4_ flux (mg C m^−2^ h^−1^) measured in light (light blue, lower panels) and dark (dark blue, upper panels) conditions depicted for each fertilization treatment. Error bars indicate SEM and light grey dots show raw data. Negative values indicate net fluxes from atmosphere to peat

CO_2_ and CH_4_ fluxes were measured, diurnal fluxes are presented in rates per day and flux rates are presented per hour. The CO_2_ flux (see Fig. 1a) measured both in dark and light conditions did not differ between treatments (Dark F_3,27_ = 0.26, p > 0.05; Light F_3,28_ = 0.33, p > 0.05; Fig. 1a). Overall, diurnal fluxes of all mesocosms showed that these are acting as net CO_2_ sinks with an average net CO_2_ intake of 2.5 ± 1.9 mg C m^−2^ day^−1^ (Table 5).

**Table 5:** Net diurnal CO_2_ flux (mg C m^−2^ day^−1^) based on a 16 h–8 h light–dark cycle

Treatment	Net diurnal CO_2_ flux (mg C m^−2^ day^−1^)	
	Mean ± SEM	n
Control	− 2298 ± 362	7
N	− 2423 ± 489	8
N + P	− 2574 ± 324	8
P	− 2606 ± 378	8

Negative numbers indicate net uptake

CH_4_ addition treatment was merged with its control, resulting in doubling of the replicates

The CH_4_ flux (see Fig. 1b) measured in light conditions was 2.2 times lower in N fertilized (N and N + P) mesocosms (resp. N 3.3 ± 0.8 mg C m^−2^ h^−1^ and NP 4.5 ± 1.0 mg C m^−2^ h^−1)^ compared to non-fertilized mesocosms (control; 9.1 ± 1.7 mg C m^−2^ h^−1^; F_3,28_ = 4.44, p = 0.01). Furthermore, the N fertilized mesocosms showed a higher CH_4_ flux in the dark (10 ± 3 mg C m^−2^ h^−1^), compared to in the light (3.3 ± 0.9 mg C m^−2^ h^−1^; F_1,48_ = 4.25, p < 0.05). The control mesocosms on the other hand showed a lower CH_4_ flux in the dark (5.1 ± 1.1 mg C m^−2^ day^−1^), compared to the light (9.1 ± 1.7 mg C m^−2^ day^−1^; see Fig. 1b), which is unlike the fertilized mesocosms. Overall, diurnal CH_4_ fluxes showed mesocosms were a net source of CH_4_, with reduced methane emission up to 30% upon N or P fertilization (Table 6).

**Table 6:** Net diurnal CH_4_ flux (mg C m^−2^ day^−1^) based on a 16 h–8 h light–dark cycle

Treatment	Net diurnal CH4 flux (mg C m^−2^ day^−1^)	
	Mean ± SEM	n
Control	192 ± 34	7
N	134 ± 29	8
N + P	147 ± 23	8
P	111 ± 26	8

Positive numbers indicate net efflux

CH_4_ addition treatment was merged with its control, resulting in doubling of the replicates

#### N_2_ fixation

**Fig. 2:**
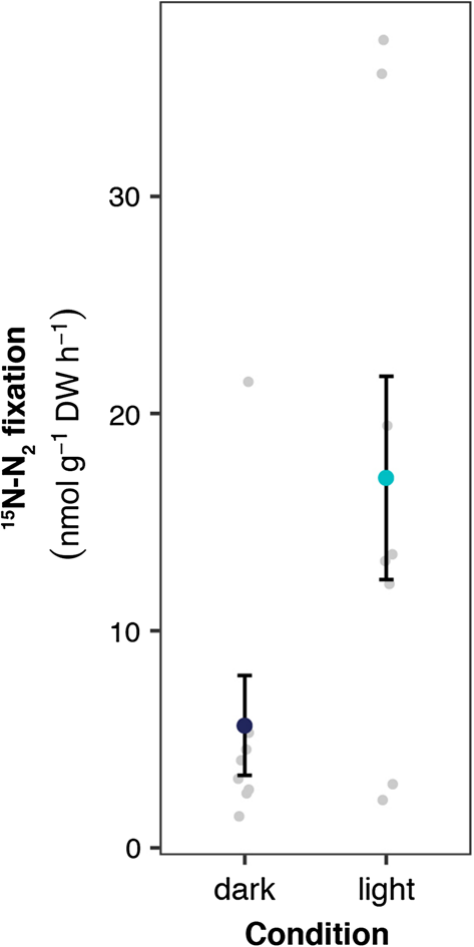
Plot showing the average N_2_ fixation rates measured as ^15^N-N_2_ incorporation from incubations performed in the field either in the dark (dark blue) or in the light (light blue). Error bars indicate SEM and light grey dots show raw data

**Fig. 3:**
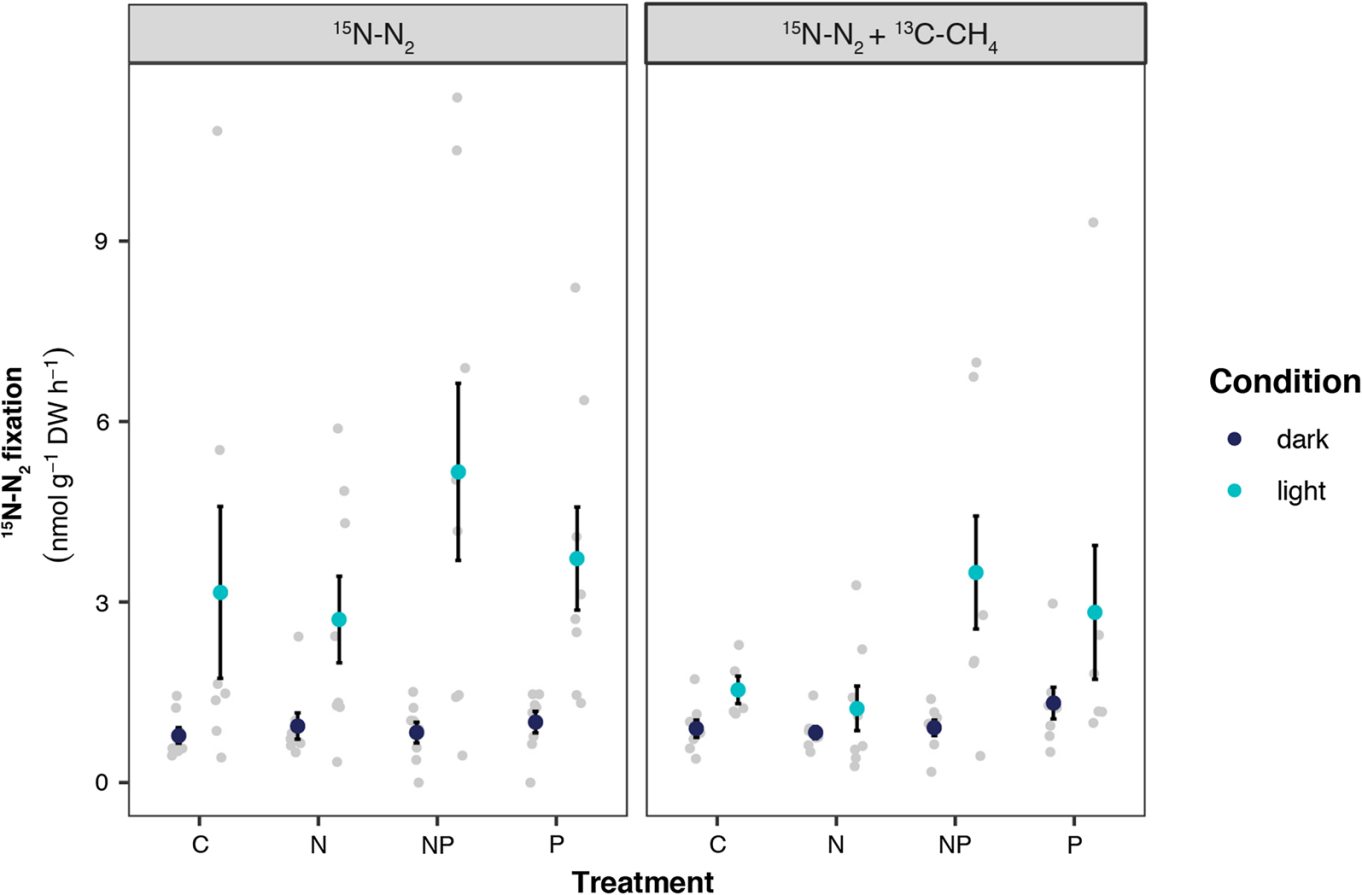
Plot showing the average N_2_ fixation rates measured as ^15^N–N_2_ incorporation for incubation with ^15^N–N_2_ (left panel) and ^15^N–N_2_ + ^13^C–CH_4_ (right panel) displayed per fertilization treatment. Incubations were performed either in the dark (dark blue) or in the light (light blue). Error bars indicate SEM and light grey dots show raw data

In the field incubation, N_2_ fixation rates were 3 times higher in the light than in the dark (resp. 409 ± 112 versus 135 ± 55 nmol N_2_ g^−1^ DW day^−1^; F_1,14_ = 5.47, p < 0.05; see Fig. 2). This effect was also observed in the mesocosm experiment (^15^N–N_2_ F_3,58_ = 28.23, p < 0.001; ^15^N–N_2_ + ^13^C–CH_4_ F_3.54_ = 12.70, p < 0.001; see Fig. 3), where both treatments (N and N + CH_4_) showed lowest N_2_ fixation rates in the dark (^15^N–N_2_: 21.3 ± 2.1 nmol N_2_ g^−1^ DW day^−1^; ^15^N–N_2_ + ^13^C–CH_4_: 23.7 ± 2.1 nmol N_2_ g^−1^ DW day^−1^) and highest rates in the light (^15^N–N_2_: 89.0 ± 14 nmol N_2_ g^−1^ DW day^−1^; ^15^N–N_2_ + ^13^C–CH_4_: 54.9 ± 10 nmol N_2_ g^−1^ DW day^−1^). None of the fertilization treatments differed from each other. Yet, it seems that mesocosms which received P and NP fertilization have higher N_2_ fixation rates on average, even though these are not significantly higher than for the N-fertilization or control.

#### CH_4_ oxidation

**Fig. 4:**
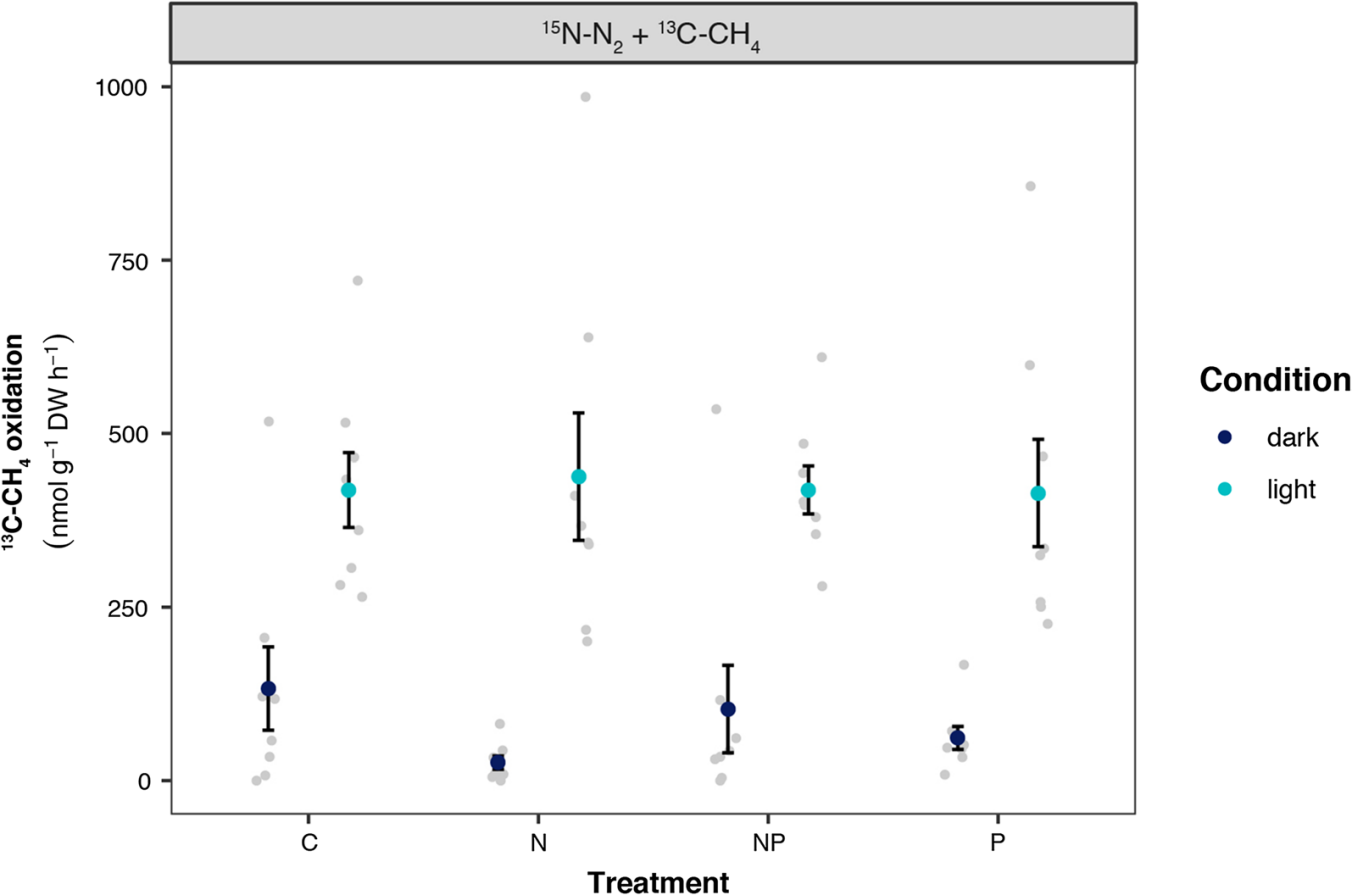
Plot showing the average CH_4_ oxidation rates measured as ^13^C–CH_4_ incorporation at the end of the experiment, displayed per fertilization treatment. Incubations were performed either in the dark (dark blue) or in the light (light blue). Error bars indicate SEM and light grey dots show raw data

CH_4_ oxidation rates as measured by ^13^C–CH_4_ incorporation, showed a highly similar pattern to N_2_ fixation rates. CH_4_ oxidation was lowest in the dark (81 ± 37 nmol CH_4_ g^−1^ DW h^−1^ Fig. 4) and five times higher in the light (423 ± 64 nmol CH_4_ g^−1^ DW h^−1^; F_1,59_ = 134.25, p < 0.001). The low CH_4_ oxidation rate in the dark correlates with high CH_4_ efflux in the dark. Similar to N_2_ fixation rates, there was no difference in CH_4_ oxidation between fertilization treatments.

## Discussion

This study aimed to investigate the effect of various potential environmental drivers on moss growth and associated microbial N_2_ fixation and CH_4_ oxidation. Hereto, we fertilized the moss (N, P and N + P) under ambient and increased CH_4_ concentrations. Additionally, the effect of light was studied. CH_4_ addition did not increase CH_4_ concentrations in the mesocosms, indicating that there was always a significant amount of CH_4_ produced by the methanogenic archaea in the sods. Although fertilization increased nutrient contents in moss tissue, no effect on *Sphagnum* moss growth was found. In the used set-up, microbial N_2_ fixation and CH_4_ oxidation were clearly positively affected by light.

### Effects of CH_4_ on N_2_ fixation rates

According to our results, it seems that internal CH_4_ production in the peat sods was already so high that the addition of CH_4_ became insignificant. Compared to other studies the concentration of CH_4_ present in the porewater of the mesocosms was already very high (491 ± 26 μmol CH_4_ L^−1^), which is similar to the reported maximum values of 407 ± 83 μmol CH_4_ L^−1^ (Kip et al. 2012) and more than ten times higher than the 41 μmol CH_4_ L^−1^ reported by Putkinen et al. (2014). Similar to CH_4_ addition in the mesocosms, N_2_ fixation was also not affected by CH_4_ addition in the isotope incubations.

### Fertilization effects

Fertilization did not affect CO_2_ fluxes, but decreased CH_4_ fluxes in the light, yet measured CH_4_ oxidation rates were not different between the different fertilization treatments. The moderate effect of N and P fertilization on nutrient content and absence of substantial effects on moss growth might indicate that 10 weeks of fertilization may have been too short to observe an effect. Therefore, a longer period of fertilization or intensified fertilization is advised for future studies and would probably have resulted in notable fertilization effects.

In addition, we supplied P to the mesocosms via injection just below the moss surface (capitula). Although this way of P-fertilization is dissimilar to other studies (Limpens et al. 2004; Fritz et al. 2012; van den Elzen et al. 2017), it resembles the way increased P fluxes enter peat ecosystems in reality. P-fertilization is in most studies sprinkled on top of the mosses or added in the inflowing surface water. These often resulted in a clear P fertilization effect (Fritz et al. 2012; van den Elzen et al. 2017). Most of the surface water entering a peatland, does not reach the capitulum level but only reaches the lower part of the moss. Furthermore, inflow of P into a fen is mostly via surface water. In order to mimick this inflow from P in our mesocosms setup, we decided to inject P instead of sprinkling it on top of the peat sods. The manner of P fertilization does seem to matter for its effect on the plant (Rydin and Clymo 1989). When P fertilization is applied on top of the capitulum more than 90% of the P will remain in the capitulum, whereas fertilization below the capitulum is translocated upwards to the stem and the capitulum and will therefore also become diluted (Rydin and Clymo 1989). If the P fertilization dilutes due to injection below the capitulum, we would have had to fertilize P for a longer period of time to observe any effect. Furthermore, by injecting P-fertilization and sprinkling N-fertilization we separated the two nutrient sources physically from each other. It might be that the physical separation also contributed to the limited fertilization effect. In addition, N and P fertilization should be repeated for a longer period to further test their effects on methane oxidation and nitrogen fixation. In order to compare the results to other studies, these two fertilizers should maybe be added in the same manner.

### Light as a key driver of N_2_ fixation

Three times higher microbial N_2_ fixation rates and five times higher CH_4_ oxidation rates in the presence of light indicate that these processes are strongly stimulated by photosynthesis, either directly or indirectly. For leguminous plant species it has recently been reported that the plants modulate their carbon allocation to symbiotic nitrogen-fixing *Rhizobia* in response to both light an N availability (Friel and Friesen 2019). For N_2_ fixation associated with *Sphagnum*, it has been observed before that N_2_ fixation rates are highest under illuminated conditions (Larmola et al. 2014; van den Elzen et al. 2017). This is true even though N_2_ fixation is inhibited by presence of O_2_ (Vitousek et al. 2013). Due to the O_2_ respiration by the moss in the dark, O_2_ availability is low in the dark (Kangas et al. 2014), hence dark conditions are in theory more beneficial for N_2_ fixation. This therefore generates two hypotheses: either N_2_ fixation activity is mainly performed by phototrophic cyanobacteria, which are most active in light conditions, or the nitrogen-fixing community is (directly or indirectly) dependent on photosynthates produced by the *Sphagnum* moss in light-conditions. The mechanisms behind this can be disentangled in future research by studying which part of the nitrogen-fixing community is active in the dark versus light by using RNA analysis on the *nifH* gene.

### Light as a key driver of CH_4_ oxidation

The net CH_4_ flux is controlled by the CH_4_ oxidation activity pattern in light and dark. The lower CH_4_ oxidation activity observed in the dark explains the higher net CH_4_ flux from the mesocosms in the dark. The light-dependency of moss-associated methanotrophy has been reported before in *Sphagnum* (Larmola et al. 2014) as well as brown-mosses (Liebner et al. 2011), and it has been reported in a Danish wetland (King 1990), but remains unexplained so-far. Furthermore, N or P fertilization seem to stimulate net CH_4_ emission in dark but reduces net CH_4_ emission in light, resulting in a lower CH_4_ efflux. The extra nutrients in the fertilized mesocosms are causing discrepancy in CH_4_ oxidation and emission and further underline the intricate role of photosynthesis in the overall CH_4_ cycle in *Sphagnum*-dominated peatlands.

We postulate four hypotheses for light-dependent CH_4_ oxidation observed here. Like N_2_ fixation, CH_4_ oxidation is either (1) directly or (2) indirectly dependent on photosynthates produced by moss (and associated photosynthetic microorganisms). In the case of indirect dependency, the photosynthates are affecting another part of the microbial community that the methanotroph subsequently benefits from (Oswald et al. 2015). Regardless of the nature of the dependency, most phototrophs (plants and *cyanobacteria*) are known to excrete photosynthetic products such as malate, citrate, glycolic acid and methanol (Tolbert and Zill 1956; Nalewajko 1966; Fall and Benson 1996; Meyer et al. 2010; Vorholt 2012). The excretion of carbohydrates is furthermore increased upon photoinhibition to dispose of electrons. Photoinhibition has been shown to occur in *Sphagnum* and is estimated to result in lowered C accumulation rates (Murray et al. 1993; Mazziotta et al. 2019). Especially in summer, in peatlands in the northern hemisphere photoinhibition might occur, because of the long days and strong light intensity.

The third (3) hypothesis is that CH_4_ oxidation is directly coupled to microbial photosynthesis (anoxygenic), which has been postulated in the 1960s (Vishniac 1960; Wertlieb and Vishniac 1967) but so far not been experimentally proven yet (Ward et al. 2019). The process has only been reported for *Rhodopseudomonas gelatinosa* (Wertlieb and Vishniac 1967) but the observed activity was low. A recent study presented metagenomic evidence for the capability of anoxygenic phototrophy by the WPS2 phylum. This phylum, without culture representatives is abundant in the *Sphagnum* microbiome (Holland-Moritz et al. 2018). Methane would be a suitable and abundant electron donor for anoxygenic phototrophy in peatland ecosystems, much more abundant than the common electron donor in anoxygenic phototrophy, which is sulfide.

The fourth (4) hypothesis is that the methanotrophs might benefit from light-dependent CH_4_ production, which has been observed in oligotrophic lakes (Grossart et al. 2011) and might as well occur in oligotrophic peatlands. This production of CH_4_ in daytime in oxygenated lakes has been postulated to be of cyanobacterial origin by cleaving methyl-phosphonates (Tang et al. 2016; Bižić-Ionescu et al. 2020; Günthel et al. 2019). In this way, methanotrophs could thrive when it’s light due to higher concentrations of both CH_4_ and O_2_ as a result of oxygenic photosynthesis. In the control treatment of our experiment, CH_4_ emission rates were indeed almost twice as high in light conditions.

Our study focused on *Sphagnum*-associated CH_4_ oxidation and N_2_ fixation, and the effects of N, P and N + P fertilization, CH_4_ addition and the additional effect of light. Light was shown to be a strong driver of both N_2_ fixation and CH_4_ oxidation, which indicates that both processes benefit directly or indirectly from photosynthesis. Leakage of photosynthates produced by the *Sphagnum* and associated photosynthetic microbial partners might be a good C-source for the organisms active in CH_4_ oxidation and N_2_ fixation. To test the various postulated hypotheses, future research should take a closer look upon the light-dependency of both CH_4_ oxidation and N_2_ fixation by measuring changes in activity based upon light on smaller timescales and intervals.

## Supplementary information

**Additional file 1.** Supplementary material containing Table S1 and Figure S1.

## Data Availability

Supplemental data are provided.
