# Supplementary material for: Microbial nitrogen fixation and methane oxidation are strongly enhanced by light in Sphagnum mosses
Source: AMB Express. 2020 Mar 31;10:61. doi: 10.1186/s13568-020-00994-9 (PMC7109220; doi:10.1186/s13568-020-00994-9)
Supplement: Supplementary file 1 — Additional file 1. Supplementary material containing Table S1 and Figure S1. [file 13568_2020_994_MOESM1_ESM.doc]

***AMB Express***

**Microbial nitrogen fixation and methane oxidation are strongly enhanced by light in *Sphagnum* mosses**

**Authors**

Martine A.R. Kox^1^, Eva van den Elzen^2^, Leon P.M. Lamers^2^, Mike S.M. Jetten^1^, Maartje A.H.J. van Kessel^1*^

**Author affiliations**

^1^ Department of Microbiology, Radboud University, Heijendaalseweg 135, 6525 AJ Nijmegen, The Netherlands

^2^ Department of Aquatic Ecology and Environmental Biology, Radboud University, Heijendaalseweg 135, 6525 AJ, Nijmegen, The Netherlands

**Corresponding Author**

^*^Corresponding Author: Maartje van Kessel

Email address: [maartje.vankessel@science.ru.nl](mailto:maartje.vankessel@science.ru.nl)

Telephone number: 0031243652952

**Additional material**

*Table S1 – Porewater pH, alkalinity and concentrations of various elements, reported for each fertilization treatment (C, N, NP and P).*

|  | **C** | | | **N** | | | **NP** | | | **P** | | |
| --- | --- | --- | --- | --- | --- | --- | --- | --- | --- | --- | --- | --- |
|  | **mean** | **±** | **sem** | **mean** | **±** | **sem** | **mean** | **±** | **sem** | **mean** | **±** | **sem** |
| **pH** | 4.78 | ± | 0.07 | 4.81 | ± | 0.07 | 4.79 | ± | 0.07 | 4.75 | ± | 0.07 |
| **alk (meq L^-1^)** | 0.36 | ± | 0.06 | 0.32 | ± | 0.06 | 0.27 | ± | 0.03 | 0.30 | ± | 0.08 |
| **PO4+ (µmol L^-1^)** | 4.49 | ± | 1.23 | 3.52 | ± | 0.89 | 4.40 | ± | 0.98 | 5.59 | ± | 1.19 |
| **NO3- (µmol L^-1^)** | 2.72 | ± | 0.52 | 3.41 | ± | 0.62 | 3.74 | ± | 0.50 | 5.14 | ± | 1.30 |
| **NH4+ (µmol L^-1^)** | 4.58 | ± | 0.86 | 5.55 | ± | 1.25 | 5.57 | ± | 1.28 | 5.84 | ± | 1.05 |
| **Cl (µmol L^-1^)** | 658.8 | ± | 75.6 | 772.1 | ± | 101.7 | 349.0 | ± | 57.3 | 644.0 | ± | 97.3 |
| **Al (µmol L^-1^)** | 6.39 | ± | 0.73 | 4.41 | ± | 0.36 | 5.08 | ± | 0.62 | 5.56 | ± | 0.87 |
| **Ca (µmol L^-1^)** | 108.0 | ± | 11.5 | 93.9 | ± | 9.41 | 86.6 | ± | 7.1 | 79.2 | ± | 7.5 |
| **Fe (µmol L^-1^)** | 243.2 | ± | 43.2 | 215.7 | ± | 38.6 | 182.2 | ± | 23.4 | 169.7 | ± | 25.0 |
| **K (µmol L^-1^)** | 218.3 | ± | 14.6 | 189.8 | ± | 16.3 | 173.7 | ± | 14.9 | 160.6 | ± | 18.7 |
| **Mg (µmol L^-1^)** | 87.05 | ± | 7.76 | 78.61 | ± | 5.59 | 77.52 | ± | 5.32 | 69.75 | ± | 5.04 |
| **Mn (µmol L^-1^)** | 4.40 | ± | 0.29 | 3.62 | ± | 0.21 | 3.75 | ± | 0.31 | 3.11 | ± | 0.22 |
| **Na (µmol L^-1^)** | 331.2 | ± | 12.0 | 334.7 | ± | 12.2 | 337.0 | ± | 11.4 | 315.0 | ± | 13.8 |
| **P (µmol L^-1^)** | 6.97 | ± | 1.46 | 6.07 | ± | 1.28 | 6.29 | ± | 1.09 | 7.71 | ± | 1.48 |
| **S (µmol L^-1^)** | 23.7 | ± | 1.5 | 22.2 | ± | 1.4 | 21.7 | ± | 1.2 | 20.7 | ± | 1.1 |
| **Si (µmol L^-1^)** | 267.2 | ± | 19.2 | 245.0 | ± | 17.1 | 243.1 | ± | 12.6 | 230.8 | ± | 13.3 |
| **Zn (µmol L^-1^)** | 0.90 | ± | 0.08 | 0.93 | ± | 0.08 | 0.89 | ± | 0.09 | 0.85 | ± | 0.09 |


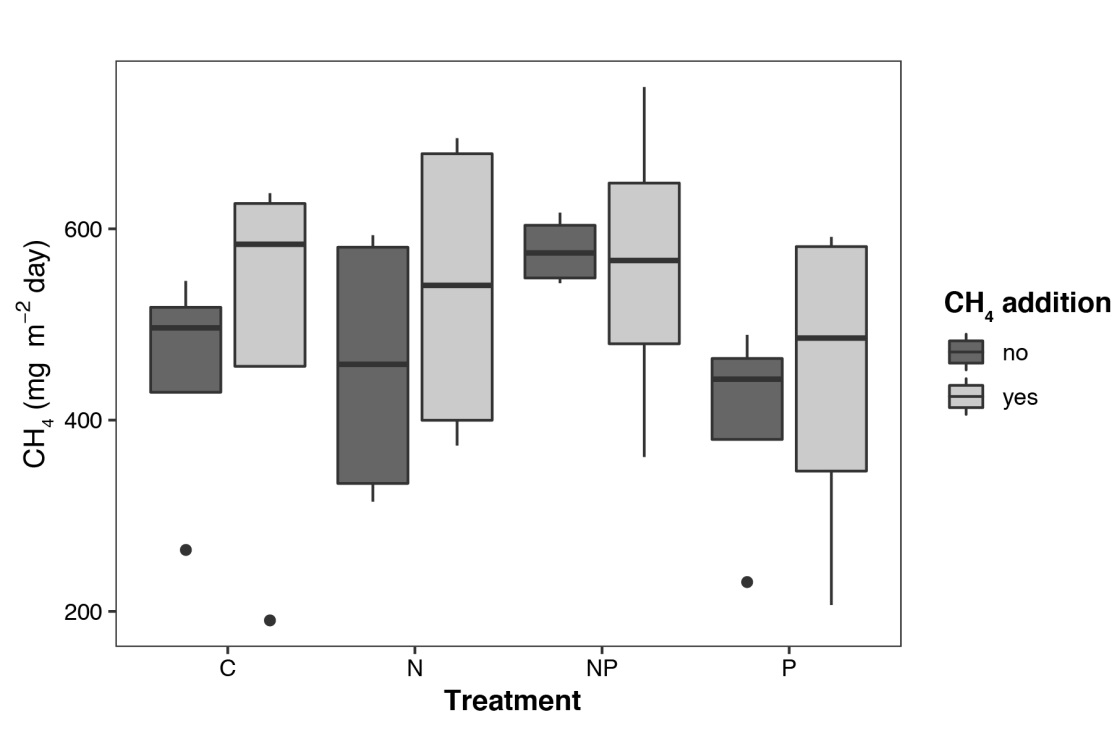


*Supplementary Fig.S1 -Dissolved CH_4_ concentration in the mesocosms per fertilization treatment with in grey the mesocosms that received CH_4_ -addition and in dark grey the no-addition mesocosms*.
